# Treatment of Neuralgia by Hypodermic Injections of Cocaine

**Published:** 1892-10

**Authors:** 


### Treatment of Neuralgia by Hypodermic Injections of Cocaine.

Malherbe, of Nantes, states (*Ther. Gazette*) that he has obtained very good results from the employment of cocaine hypodermically, for neuralgia, in one of the following formulæ:

| | | | |
|---|---|---|---|
| | R | Hydrochlorate of cocaine, - | gr. xv. |
| | | Water, - - - . - | oz. vi. |
| | M. | | |
| Or, | R | Hydrochlorate of cocaine, - | gr. xv. |
| | | Boric acid, - - - - - | gr. viij. |
| | | Distilled water, - - - | oz. vj. |
| | M. | | |
| Or, | R | Neutral glycerin, - - - | oz. j to ij. |
| | | Hydrochlorate of cocaine, - | gr. xv. |
| | M. | | |

In making these solutions it is necessary that the cocaine should be absolutely pure. If it is used externally over the nerve it may be dissolved in liquid vaseline in the proportion of two per cent. The method which Malherbe employs is to raise a small portion of the skin immediately over the seat of pain, and to inject underneath it a drop of the anæsthetic. The tender nerve is found by the pain which is elicted by gently moving the needle about in the tissues which have been made anæsthetic by the first drop which has been inserted by the needle, when several more drops are traced directly in contact with the nerve fibre. Notwithstanding the rapid absorption of cocaine in the blood-vessels, this writer has not seen the untoward effects which others have observed in the hypodermic injection of these drugs, particularly when used about the head. It was found also that larger doses could be used after the drug had been given for a number of days, the patient becoming immune to the smaller doses.

